# NodeFlow: Towards End-to-End Flexible Probabilistic Regression on Tabular Data

**DOI:** 10.3390/e26070593

**Published:** 2024-07-11

**Authors:** Patryk Wielopolski, Oleksii Furman, Maciej Zięba

**Affiliations:** 1 Department of Artificial Intelligence, Wrocław University of Science and Technology, 50-370 Wrocław, Poland; 2 Tooploox Ltd., 53-601 Wrocław, Poland

**Keywords:** probabilistic regression, tabular data, normalizing flows, decision tree ensembles, neural decision tree

## Abstract

We introduce NodeFlow, a flexible framework for probabilistic regression on tabular data that combines Neural Oblivious Decision Ensembles (NODEs) and Conditional Continuous Normalizing Flows (CNFs). It offers improved modeling capabilities for arbitrary probabilistic distributions, addressing the limitations of traditional parametric approaches. In NodeFlow, the NODE captures complex relationships in tabular data through a tree-like structure, while the conditional CNF utilizes the NODE’s output space as a conditioning factor. The training process of NodeFlow employs standard gradient-based learning, facilitating the end-to-end optimization of the NODEs and CNF-based density estimation. This approach ensures outstanding performance, ease of implementation, and scalability, making NodeFlow an appealing choice for practitioners and researchers. Comprehensive assessments on benchmark datasets underscore NodeFlow’s efficacy, revealing its achievement of state-of-the-art outcomes in multivariate probabilistic regression setup and its strong performance in univariate regression tasks. Furthermore, ablation studies are conducted to justify the design choices of NodeFlow. In conclusion, NodeFlow’s end-to-end training process and strong performance make it a compelling solution for practitioners and researchers. Additionally, it opens new avenues for research and application in the field of probabilistic regression on tabular data.

## 1. Introduction

Tabular regression involves predicting a continuous target variable based on structured data arranged in a tabular format. It is a vital task in machine learning with applications in various domains, including finance, healthcare, and marketing. In these domains, making reliable and informed decisions is of utmost importance due to potential consequences or impacts and requires not only accurate predictions but also robust uncertainty quantification. These kinds of properties can be obtained by the usage of probabilistic methods that go beyond point estimation by modeling the entire conditional distribution. This approach offers several advantages, including the ability to quantify uncertainty, capture complex data distributions, and provide a more comprehensive understanding of the data.

Regarding deterministic tabular regression, there have been two distinct paths of research in the field of regression on tabular data without any clear conclusion of the best approach to the problem [1,2]. The first path focuses on gradient-boosted trees, exemplified by popular approaches such as XGBoost [3], CatBoost [4], and LightGBM [5]. These methods have demonstrated remarkable performance in point estimation tasks, leveraging ensemble techniques to capture complex relationships in the data. The second research path explores deep learning techniques for regression on tabular data with models such as NODE [6], TabNet [7], or FT-Transfomer [8]. These methods, with their ability to capture intricate patterns and relationships, have shown promise in surpassing the performance of gradient-boosted trees. They offer flexibility in handling various data types, including categorical variables, and can capture complex interactions among features. However, challenges specific to tabular data, such as feature interactions and interpretability, continue to be active research areas.

In the context of probabilistic tabular regression, recent research predominantly centers on expanding tree-based methods. The development of the new methods has resulted in models such as NGBoost [9], PGBM [10], and a probabilistic extension of CatBoost [11]. However, these methods are predominantly based on parametric distributions, with CatBoost limited to modeling only Gaussian distributions. As a result, a pressing need remains for more flexible approaches that can accurately capture a broader range of complex data distributions encountered in practical scenarios. The recent work on TreeFlow [12] showed that combining tree-based methods with normalizing flows can improve the modeling capabilities; however, a lack of end-to-end optimization might lead to suboptimal results.

To overcome the limitations associated with the absence of end-to-end optimization, we propose NodeFlow, a novel framework for flexible probabilistic regression on tabular data. NodeFlow combines the advantages of tree-based structures, deep learning approaches, and normalizing flows to provide an accurate probabilistic regression approach that can be learned end to end. By combining Neural Oblivious Decision Ensembles (NODEs) and Conditional Continuous Normalizing Flows (CNFs), NodeFlow offers a unique solution that enables the modeling of complex data distributions encountered in probabilistic tasks. Through extensive evaluations and comparative studies on benchmark datasets, we demonstrate the effectiveness of NodeFlow in capturing the underlying data distributions and providing state-of-the-art results for multivariate probabilistic regression problems and competitive performance in univariate regression tasks.

Concluding, our contributions are as follows:

- We introduce NodeFlow, to the best of our knowledge, the first framework to apply an end-to-end, tree-structured deep learning model for probabilistic regression on tabular data;
- We demonstrate NodeFlow’s superior performance in multivariate probabilistic regression and competitive results in univariate tasks on benchmark datasets, establishing its effectiveness;
- We conduct a focused ablation study, hyperparameter sensitivity analysis, and computational efficiency assessment, validating NodeFlow’s design and scalability.

## 2. Literature Review

### 2.1. Tree-Based Regression on Tabular Data

Standard tree-based regression approaches, including XGBoost [3], CatBoost [4], and LightGBM [5], have emerged as state-of-the-art methods for modeling tabular data in regression problems. These frameworks leverage ensemble techniques and advanced optimizations to achieve remarkable performance in various domains. XGBoost is an optimized gradient-boosting framework that combines decision trees to capture complex relationships in tabular data. CatBoost incorporates novel techniques to handle categorical features effectively, while LightGBM utilizes tree-based learning algorithms and efficient data processing strategies. Their widespread adoption and success in diverse applications highlight their effectiveness and prominence in the field of tabular regression modeling, enabling accurate point estimation and capturing intricate patterns within the data.

### 2.2. Tree-Based Probabilistic Regression on Tabular Data

In recent years, several approaches have been developed for probabilistic regression on tabular data, including NGBoost [9], CatBoost with univariate Gaussian support [11], and the Probabilistic Gradient Boosting Machine (PGBM) [10], each offering unique methods to model probabilistic distributions and improve regression performance. NGBoost is a versatile algorithm that can model various probabilistic distributions using a defined probability density function. It estimates distribution parameters by optimizing scoring rules such as the negative log-likelihood (NLL) or Continuous Ranked Probability Score (CRPS). RoNGBa [13] is an NGBoost extension that enhances performance through improved hyperparameter selection. CatBoost, a gradient-boosting framework, has also been adapted to probabilistic regression but supports only univariate Gaussian distributions. PGBM treats leaf weights as random variables and can model different posterior distributions, albeit limited to location and scale parameters.

### 2.3. Deep Learning Regression on Tabular Data

In recent years, deep neural networks have achieved remarkable success in handling unstructured data, but their effectiveness in dealing with tabular data remains inconclusive. Several research papers, including [6,7,8,14,15], have introduced new deep learning regression methods that demonstrate superiority over tree-based methods. However, recent surveys have produced conflicting results on this topic. Notably, Borisov et al. [1] conducted a study comparing deep models to traditional machine learning methods on selected datasets. They found that deep models consistently outperformed traditional methods, but no single deep model universally outperformed all others. These findings highlight the nuanced performance of deep learning models on tabular data. Additionally, recent benchmarks conducted by Grinsztajn et al. [2] compared tree-based models and deep learning methods, specifically on tabular data. The benchmarks revealed that tree-based models such as XGBoost and random forests remain state-of-the-art for medium-sized datasets (with fewer than 10,000 samples). Notably, even without considering their superior processing speed, tree-based models maintained a competitive edge over deep learning approaches.

Neural Oblivious Decision Ensembles (NODEs), introduced by [6], are a deep learning architecture that extends ensembles of oblivious decision trees. It combines end-to-end gradient-based optimization with multi-layer hierarchical representation learning. DNF-Net, proposed by [7], is a neural architecture incorporating a disjunctive normal form (DNF) structure, allowing efficient and interpretable feature selection. It promotes localized decisions over small feature subsets, enhancing interpretability and mitigating overfitting. TabNet [14] is a deep learning architecture specifically tailored for tabular data. It processes raw tabular data without preprocessing, facilitating seamless integration into end-to-end learning. Sequential attention mechanisms identify crucial features at each decision step, enhancing interpretability and learning efficiency. TabNet also provides interpretable feature attributions and insights into the model’s global behavior. Gorishniy et al. [8] proposed FT-Transformer, a modified version of the Transformer architecture designed for tabular data. FT-Transformer incorporates both categorical and continuous features, employs self-attention mechanisms to capture feature relationships, and integrates residual connections akin to ResNet. In addition to these approaches, SAINT (Self-Attention and Intersample Attention Transformer) [15] is a hybrid deep learning approach designed to solve tabular data problems. SAINT integrates attention over both rows and columns, an enhanced embedding method, and a contrastive self-supervised pre-training technique.

### 2.4. Deep Learning Probabilistic Regression on Tabular Data

Recently, there has been limited research on Probabilistic Deep Learning for tabular data. One notable method in this area is Deep Ensemble [16], which involves training an ensemble of neural networks using negative log-likelihood optimization with a Gaussian distribution as the modeling choice. The authors also incorporate adversarial training to produce smoother predictive estimates. Another approach, MC-Dropout [17], extends the use of dropout to capture model uncertainty during inference. By sampling multiple dropout masks during inference and averaging the predictions over these masks, an ensemble of models is created to capture model uncertainty collectively. Probabilistic Backpropagation [18] treats the neural network weights as random variables and approximates their posterior distribution using a factorized Gaussian distribution. This approximation is updated iteratively utilizing a combination of variational inference and stochastic gradient descent. More recently, TreeFlow [12] introduced a tree-based approach that combined the advantages of tree ensembles with the flexibility of modeling probability distributions using normalizing flows. By using a tree-based model as a feature extractor and combining it with a conditional variant of normalizing flow, TreeFlow enabled the modeling of complex distributions in regression outputs. While TreeFlow has shown superior performance in some cases, its lack of end-to-end training may result in suboptimal results.

In conclusion, the existing methods for probabilistic regression on tabular data often have limitations in terms of their modeling flexibility or end-to-end training. NodeFlow addresses these limitations by combining the tree-based NODE with the flexibility of CNFs, offering end-to-end training and a unique solution for probabilistic regression on tabular data.

## 3. NodeFlow

**Figure 1:**
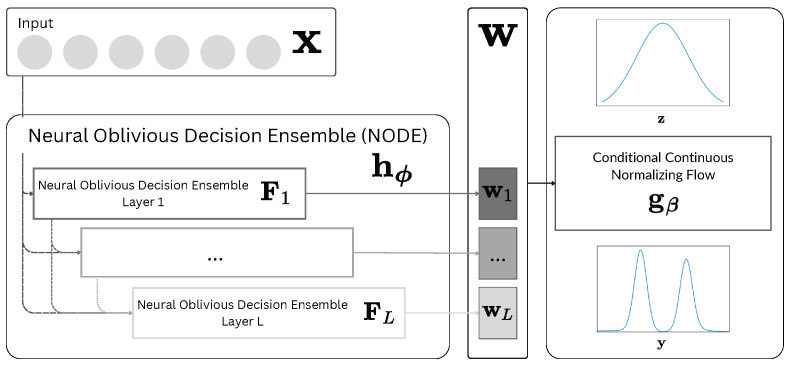
Architectural overview: NodeFlow leverages a Neural Oblivious Decision Ensemble (NODE) to process the input vector, extracting a hierarchical representation. This representation conditions a Continuous Normalizing Flow (CNF), enabling the flexible modeling of the probabilistic distribution of the multidimensional response vector.

**Figure 2:**
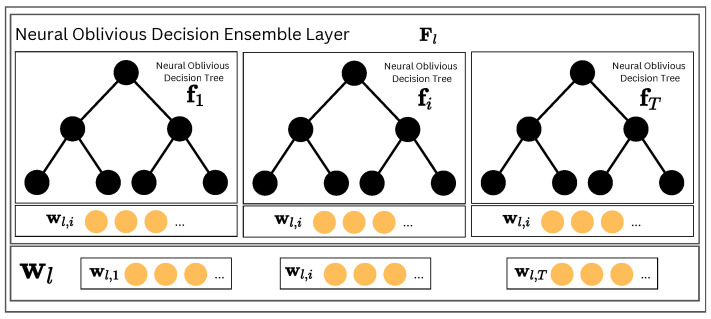
The Neural Oblivious Decision Ensemble (NODE) layer is a key component of NodeFlow’s architecture. It comprises several Neural Oblivious Decision Trees, each generating a multidimensional output vector. These vectors are then combined through concatenation to produce the final output of the NODE Layer.

The architecture of NodeFlow is provided in Figure 1. The real-valued input vector x of dimensionality *D* is initially processed using a Neural Oblivious Decision Ensemble, consisting of NODE Layers (details of the layer are depicted in Figure 2) arranged in a multi-layer hierarchical structure. It allows the extraction of rich hierarchical representation w. We use that vector as a conditioning factor for the conditional Continuous Normalizing Flow (CNF) in the next step. This component is responsible for the flexible modeling of the conditional probabilistic distribution of vector y. It is worth mentioning that there are no restrictions on the response vector dimensionality. Thus, we could cover both uni- and multivariate regression problems. The whole architecture is trained in an end-to-end fashion using gradient-based optimization.

### 3.1. Extracting Hierarchical Representation with NODE

In order to extract a rich hierarchical representation for a given input x, we utilize Neural Oblivious Decision Ensemble (NODE) $h_{\phi }\left(x\right)$ parametrized by ϕ, which is a machine learning architecture that combines differentiable oblivious decision trees f(x) (ODTs). In this section, we start by introducing the ODTs. Then, we discuss the composition of the ODTs into the NODE Layer, and finally, we present the NODE component responsible for the hierarchical representation extraction in NodeFlow.

A single differentiable oblivious decision tree f(x) of depth *d* is defined as:

(1) $$f\left(x\right)=\sum_{j=1}^{2^{d}}r_{j}\cdot l_{j}\left(x\right),$$

where $r=[r_{1},\cdots ,r_{2^{d}}]$ is a $2^{d}$-dimensional vector of real-valued trainable responses for each of the considered leaves in the tree, and $l\left(x\right)=[l_{1}\left(x\right),\cdots ,l_{2^{d}}\left(x\right)]$ is a $2^{d}$-dimensional vector of real-valued entries from the range [0,1]. The vector is called a “choice vector” and corresponds to the probability of the sample ending up in the specific leaf.

To compute the choice vector, it is requisite to perform a multiplication of the probabilities associated with selecting either the left or right path across successive depth levels within the tree structure. It is important to note that in an oblivious decision tree, only one decision is made at each level of depth, which is referred to as $c_{i}\left(x\right)$ at depth *i*. The final choice vector l is derived using the formula:

(2) $$l\left(x\right)=\left[\begin{array}{c} c_{1}\left(x\right)\\ 1-c_{1}\left(x\right) \end{array}\right]⊗\left[\begin{array}{c} c_{2}\left(x\right)\\ 1-c_{2}\left(x\right) \end{array}\right]⊗\cdots ⊗\left[\begin{array}{c} c_{d}\left(x\right)\\ 1-c_{d}\left(x\right) \end{array}\right],$$

where ⊗ denotes the Kronecker product.

To ensure differentiability during training in the tree split, we utilized the α-entmax function [19], which generalizes the Softmax (α=1) and Sparsemax (α=2) functions and allows for the learning of sparse choices through gradient-based learning methods. The feature choice function $c_{i}\left(x\right)$ is then calculated as a two-class entmax function over the transformed output of the feature selection function $k_{i}\left(x\right)$. This can be expressed formally as:

(3) $$c_{i}\left(x\right)={\mathrm{entmax}}_{\alpha }\left([\frac{k_{i}\left(x\right)-b_{i}}{\tau_{i}},0]\right)$$

where $b_{i}$ and $\tau_{i}$ are learnable threshold and scale parameters, and α is the entmax function’s hyperparameter that controls the level of “sparsity” in the output. In addition, the function for selecting differentiable features can be written as follows:

(4) $$k_{i}\left(x\right)=\sum_{j=1}^{D}x_{j}\cdot p_{j}^{\left(i\right)},$$

where $p^{\left(i\right)}$ is the *D*-dimensional vector of feature selection weights given by the formula $p^{\left(i\right)}={\mathrm{entmax}}_{\alpha }\left(F_{i,\cdot }\right)$. Moreover, $F\in R^{d\times D}$ is called the feature selection matrix, and it is a real-valued, learnable matrix.

In summary, the differentiable oblivious decision tree, denoted as f, is parameterized by the response vector r, threshold values τ, scale factors b, and the feature selection matrix F, facilitating gradient-based learning.

To form the Neural Oblivious Decision Ensemble layer $F_{l}$ (depicted in Figure 2), we need to concatenate all outputs of the *T* individual $f_{1},\ldots ,f_{T}$ ODTs forming the layer. The final output can be written as

(5) $$F_{l}\left(\cdot \right)=\left[f_{1}\left(\cdot \right),\ldots ,f_{T}\left(\cdot \right)\right].$$

Finally, the NODE architecture $h_{\phi }\left(x\right)$ is composed of *L* stacked NODE layers in a similar fashion to the DenseNet model. It means that each layer takes the concatenated outputs of all previous layers as input, allowing the model to learn both low-level and high-level features. It can be written as:

(6) $$w_{0}=x;\;\forall_{l\in [1,L]}\;w_{l}=\left[F_{l}\left(w_{l-1}\right),w_{l-1}\right].$$

The outputs from each layer are concatenated to create the final representation extracted using NODE, $w=\left[w_{1},\ldots ,w_{L}\right]=h_{\phi }\left(x\right)$. The representation w is further delivered to CNFs as a conditioning factor.

### 3.2. Probabilistic Modeling with CNFs

We consider the conditional variant of CNFs provided in [20,21], where the conditional factor $w=h_{\phi }\left(x\right)$ is delivered to the function of the dynamics of z(t), $g_{\beta }\left(z\left(t\right),t,w\right)$, parametrized by β. In the CNF setting, we aim at finding a solution $y:=z(t_{1})$ for the differential equation, assuming the given initial state $z:=z(t_{0})$ with a known prior, where z is a random variable, $z(t_{0})$ is a base distribution, and $z(t_{1})$ constitutes our observable data. Moreover, $t_{0}$ and $t_{1}$ denote the start and end points, respectively, of the continuous transformation process. The transformation function between y and z is represented as:

(7) $$y=u_{\beta ,\phi }\left(z,x\right)=z+\int_{t_{0}}^{t_{1}}g_{\beta }\left(z\left(t\right),t,h_{\phi }\left(x\right)\right)dt.$$

The inverse form of the transformation $u_{\beta ,\phi }\left(\cdot \right)$ is given by equation:

(8) $$z=u_{\beta ,\phi }^{-1}\left(y,x\right)=y-\int_{t_{0}}^{t_{1}}g_{\beta }\left(z\left(t\right),t,h_{\phi }\left(x\right)\right)dt.$$

Finally, we can calculate the log-probability of target variable y given the vector of features x by the following formula:

(9) $$\log p\left(y|x\right)=\log p\left(z\right)-\int_{t_{0}}^{t_{1}}\mathrm{Tr}\left(\frac{\partial g_{\beta }\left(z\left(t\right),t,h_{\phi }\left(x\right)\right)}{\partial z(t)}\right)dt,$$

which can be solved analogously to FFJORD [22] by employing the adjoint method to backpropagate through the solution of the neural ODE.

### 3.3. Training NodeFlow

Using the formula (9) that directly defines log-probability, we can train NodeFlow by directly optimizing the negative log-likelihood function. Let us assume we are given a dataset $D={\left(x_{n},y_{n}\right)}_{n=1..N}$, where $x_{n}=\left(x_{n}^{1},\ldots ,x_{n}^{D}\right)$ represents a *D*-dimensional random feature vector, and $y_{n}=\left(y_{n}^{1},\ldots ,y_{n}^{P}\right)$ is the *P*-dimensional vector of targets. The training of the probabilistic model involves minimizing the conditional negative log-likelihood function (NLL), defined as:

(10) $$Q\left(\beta ,\phi \right)=-\sum_{n=1}^{N}\log p(y_{n}|x_{n},\beta ,\phi ).$$

The goal during the training process is to find the optimal parameters $\beta^{*}$ and $\phi^{*}$ such that:

(11) $$\beta^{*},\phi^{*}=\underset{\beta ,\phi }{arg min}Q\left(\beta ,\phi \right).$$

All model parameters β,ϕ are trained end to end by optimizing the above-mentioned NLL using the standard gradient-based approach. Such an approach simplifies the modeling process by allowing the entire model to be trained using a single optimization algorithm. Moreover, the model can automatically learn relevant hierarchical representations of the data directly from the raw input data, capturing both low-level and high-level features. This eliminates the need for manual feature engineering, which can be time-consuming and require domain expertise.

## 4. Experiments

In this section, we present a comprehensive set of experiments to evaluate the performance and effectiveness of NodeFlow in the context of tabular regression problems. We aimed to assess NodeFlow’s capabilities in capturing complex data distributions, generating accurate point estimates, and quantifying uncertainty. To achieve this, we conducted evaluations on univariate and multivariate benchmark datasets, comparing NodeFlow with other reference methods. We measured the performance using various evaluation metrics such as the negative log-likelihood (NLL), Continuous Ranked Probability Score (CRPS), and Root-Mean-Square Error (RMSE). Through these experiments, we aimed to demonstrate the performance and flexibility of NodeFlow in probabilistic regression tasks, contributing to the advancement of the field and providing insights for practical applications.

### 4.1. Methodology

In our evaluation, we adhered to the established probabilistic regression benchmark, as delineated in previous studies [9,11,12], excluding the Boston dataset in consideration of ethical concerns [23]. For univariate regression, we employed nine datasets from the UCI Machine Learning Repository and six datasets for multivariate regression as suggested by [12], with comprehensive dataset details provided in the Appendix A. In alignment with protocols from the referenced literature, we generated 20 random folds for the univariate regression datasets (with the exception of Protein at five folds and Year MSD at a single fold), designating 10% of the data for testing in each fold. The remainder was divided into an 80%/20% training/validation split for epoch selection. Our results are presented as the mean and standard deviation across validation folds. We benchmarked NodeFlow against a suite of models, including four tree-based probabilistic models (NGBoost, RoNGBa, CatBoost, PGBM), a deep learning approach (Deep Ensemble), and a hybrid model (TreeFlow) for univariate tasks. For multivariate regression challenges, we adopted training/testing splits as per the referenced protocols, comparing NodeFlow against NGBoost variants and TreeFlow. The architecture specifics and hyperparameter tuning methodology for NodeFlow are detailed in the Appendix B.

### 4.2. Probabilistic Regression Framework

This segment evaluates NodeFlow’s performance within a probabilistic framework, analyzing its negative log-likelihood (NLL) scores against benchmark datasets for both univariate and multivariate regression tasks previously outlined.

In Table 1, we present the evaluation results for the univariate regression task, where NodeFlow exhibited competitive performance across a range of datasets, frequently achieving the best or second-best NLL scores. Notably, NodeFlow excelled on the Year MSD dataset and secures commendable second-best results on the Wine, Protein, Power, and Kin8nm datasets. Our analysis extended to a detailed comparison of NodeFlow against various methodological approaches, including deep learning-based methods, tree-based ensemble methods, and the hybrid method TreeFlow. Against the Deep Ensemble, NodeFlow consistently demonstrated superior or at least equivalent performance, with particularly noteworthy achievements on the Energy, Power, Protein, Wine, and Yacht datasets. This is especially significant for the Protein and Wine datasets, which are characterized by their underlying multimodal target distributions—a scenario where NodeFlow’s capabilities of flexible distribution modeling were especially advantageous (refer to [12] for details). When compared to tree-based methods such as CatBoost, NGBoost, RoNGBa, and PGBM, NodeFlow maintained a competitive edge, often outperforming or matching the best results, underscoring its robust ability to model complex data relationships within tabular datasets. In direct comparison with TreeFlow, NodeFlow and TreeFlow exhibited closely matched performance, with each method surpassing the other under different circumstances. This comparative analysis not only highlights NodeFlow’s versatile efficacy across a broad spectrum of univariate regression challenges but also its capacity to address the intricacies of tabular data modeling through its advanced, adaptive learning framework.

In Table 2, we detail NodeFlow’s performance across multivariate probabilistic regression tasks, where it consistently outperformed competing approaches in five of the six datasets examined. Compared with TreeFlow, NodeFlow’s superiority was particularly evident in datasets with multiple target dimensions, such as scm20d (16 target dimensions) and Energy (17 target dimensions). For two-dimensional target datasets like Parkinsons and US Flight, NodeFlow continued to outperform, albeit with a narrower margin. The distinction became more nuanced with one-dimensional targets, as presented in prior analyses, where NodeFlow and TreeFlow showed competitive yet comparable results. This differentiation underscores the strength of NodeFlow’s end-to-end learning model, which excels in complex, high-dimensional settings by providing finely tuned representations. Such comprehensive learning is absent in TreeFlow, limiting its effectiveness in comparison. This evidence reinforces the indispensable value of end-to-end learning in achieving optimal performance, particularly in addressing the intricate demands of multivariate regression problems.

### 4.3. Point-Prediction Regression Setup

This section assesses the effectiveness of our method in a point-prediction context by comparing its Root-Mean-Square Error (RMSE) scores on the univariate regression datasets. To calculate the RMSE results for the TreeFlow and NodeFlow methods, we used the RMSE@K metric introduced in [12], where K=2. This metric is suitable for uni- and multivariate regression problems with multiple-point predictions. We present the results in Table 3. Our method achieved the best results on two datasets and ranked second on two others. For the remaining datasets, it remained competitive with benchmark methods. Notably, these results are commendable, considering our approach is designed for probabilistic setups. Providing point estimates, particularly from multimodal distributions, presents unique challenges compared to simply taking the mean of parametric distributions like Gaussian. This context underscores the strength of our method’s performance across various datasets.

### 4.4. Summary

In summary, our evaluation of NodeFlow across both probabilistic and point-prediction scenarios demonstrates its efficacy. While NodeFlow’s performance on tasks with one-dimensional targets aligns with existing benchmarks, it distinctly excels in handling problems with two or more target dimensions. The results unequivocally indicate that the greater the dimensionality of the target variable, the more pronounced NodeFlow’s superiority becomes. This superior performance is attributed to NodeFlow’s flexible probabilistic modeling and comprehensive end-to-end learning approach, ensuring highly tailored representations for complex problems. Consequently, NodeFlow stands out as a superior method for probabilistic regression tasks involving high-dimensional targets, affirming its suitability for addressing advanced modeling challenges.

## 5. Ablation Studies

In the pursuit of a comprehensive understanding of NodeFlow method, a series of ablation studies were undertaken to scrutinize the impacts of critical design choices therein. Specifically, this investigation focused on two integral constituents: the feature representation component, in NodeFlow attained by the usage of NODEs, and the probabilistic modeling segment, which was realized through the utilization of CNFs. We evaluated our methods using both probabilistic and point-prediction frameworks. Additionally, we conducted a qualitative analysis of the learned representations and estimated probability density functions. Moreover, the results of the computational time comparison are included in Section 6.

### 5.1. Feature Representation Component

In our ablation study, we assessed the critical role of the Neural Oblivious Decision Ensemble (NODE) component in enhancing feature extraction within our proposed framework, NodeFlow. To this end, we conducted both quantitative and qualitative analyses, employing two benchmarking variants for comparison: one with the NODE component removed, relying solely on min-max scaling (termed as *CNF*), and another replacing the NODE with a shallow Multilayer Perceptron (MLP), labeled as *CNF + MLP*.

Quantitative results, detailed in Table 4, evaluate the performance across probabilistic and point-prediction metrics: negative log-likelihood (NLL), Continuous Ranked Probability Score (CRPS), and Root-Mean-Square Error at 2 (RMSE@2), presented as mean values alongside their standard deviations. The experimental setup was kept consistent with the main experiments.

Our findings reveal that NodeFlow, with the NODE component integrated, consistently delivered the lowest NLL values across a majority of datasets, highlighting its exceptional data modeling and prediction accuracy capabilities. Additionally, NodeFlow surpassed comparative approaches in CRPS, indicating its enhanced precision in probabilistic forecasting. Furthermore, NodeFlow achieved the most favorable RMSE scores, underlining the NODE component’s pivotal role in achieving precise point predictions.

**Figure 3:**
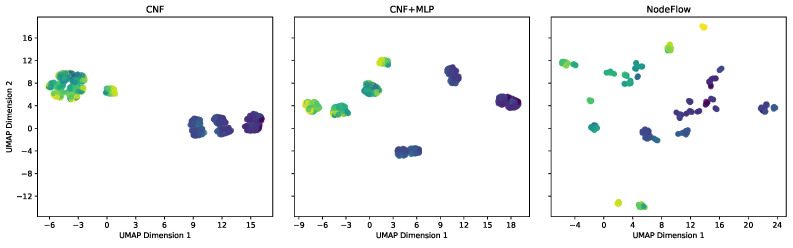
Feature representations for the Energy dataset via UMAP for the ablation study. Left: CNF model, showing rescaled data within (−1,1). Center: CNF + MLP model, indicating improved structuring. Right: NodeFlow with NODE, illustrating the superior hierarchical organization. Points are color-coded by the target variable.

In our qualitative analysis, we visualized feature representations derived from the models, utilizing dimensionality reduction via the UMAP algorithm [24] and color-coding each point according to its target variable. Figure 3 illustrates these representations for the Energy dataset. The leftmost visualization corresponds to the CNF model, which, lacking additional processing layers, essentially reflects the rescaled raw dataset within the (−1,1) range. The middle image depicts the representation from the CNF + MLP model, while the rightmost image shows the outcome of employing a NODE within the NodeFlow method. Comparatively, the NodeFlow method’s representation, facilitated by NODE processing, showcases a significantly enhanced separation and disentanglement of observations, with distinct clusters forming around similar target values. This level of disentanglement, absent in the CNF models’ representations, likely plays a crucial role in NodeFlow’s superior performance across quantitative metrics.

Collectively, these outcomes validate the NODE component’s indispensable contribution to NodeFlow’s architecture, ensuring competitive or superior performance in NLL, CRPS, and RMSE metrics and disentangled and more clearly separated representations compared to the alternatives examined.

### 5.2. Probabilistic Modeling Component

In this ablation study, we evaluated the effectiveness and fit of the probabilistic modeling component within our framework. Specifically, we substituted the CNF component with standard probabilistic distributions, labeling these variants as *NodeGauss* (using a Gaussian distribution) and *NodeGMM* (employing a mixture of Gaussians). This experimental design mirrors the setup of our previous ablation studies.

The findings, detailed in Table 5, indicate that NodeFlow consistently surpassed both NodeGauss and NodeGMM in the negative log-likelihood (NLL) across the majority of the datasets, with NodeGMM outperforming NodeFlow only in a single dataset instance. In terms of the Continuous Ranked Probability Score (CRPS), NodeFlow attained the lowest scores universally, indicating a more accurate calibration of predictive uncertainty relative to the alternatives. Point-prediction results further underscored NodeFlow’s superiority as the most effective approach. Notably, these outcomes underscored the benefit of integrating a versatile probabilistic modeling component, as evidenced by the enhanced performance across all evaluated metrics.

**Figure 4:**
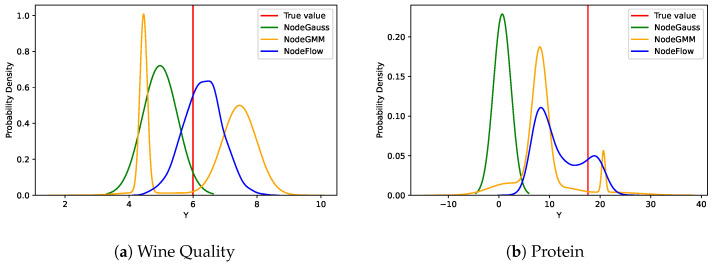
Comparison of probability density functions estimated by NodeFlow, NodeGauss, and NodeGMM for selected samples from the Wine Quality and Protein datasets.

Figure 4 illustrates the probability density functions estimated by NodeFlow, NodeGauss, and NodeGMM for selected samples from the Wine Quality and Protein datasets. These datasets were chosen due to their complex distributions and the significant differences in results among the models. In the Wine Quality example, NodeFlow produced a distribution concentrated between values six and seven, lacking the distinct peak characteristic of Gaussian distributions. The Protein dataset example showcased NodeFlow’s ability to model a bimodal distribution with significant probability mass between peaks and a heavy right tail. Notably, both NodeGauss and NodeGMM struggled to fully capture the complexity of these sample distributions. This observation underscored the necessity for more sophisticated distributional modeling, as provided by our Conditional Normalizing Flow (CNF) component in NodeFlow.

Overall, NodeFlow’s uniform advantage across diverse metrics and datasets together with supporting visualizations robustly validates the integral role of the CNF component in its architecture, underscoring its indispensability for achieving optimal model performance.

## 6. Computational Time Comparison

In this analysis, we evaluated the training duration of NodeFlow relative to benchmark models from ablation studies, including CNF, CNF + MLP from the feature representation study, and NodeGauss and NodeGMM from the probabilistic modeling investigation. Our objective was to elucidate the computational demands of training each model across various datasets, as detailed in Table 6. The table delineates the mean training times and their standard deviations, offering insights into both average performance and variability.

In the feature representation study, the marginal difference in training times among NodeFlow, CNF, and CNF + MLP suggests that the NODE component’s integration is cost-effective, enhancing the model output without a corresponding surge in training duration. Conversely, the probabilistic modeling study indicates a more pronounced disparity in training times, particularly between NodeFlow and the NodeGauss and NodeGMM variants, with NodeFlow achieving superior results with a proportional increase in computational time.

Overall, NodeFlow presents itself as a robust solution for probabilistic regression tasks on tabular data, adeptly balancing efficiency in training time with excellence in performance. This equilibrium makes NodeFlow a compelling option for both academic research and practical implementation, highlighting its potential as a preferred method in the domain.

## 7. Conclusions

In this study, we introduced NodeFlow, a novel framework for probabilistic regression on tabular data, leveraging Neural Oblivious Decision Ensembles (NODEs) and Conditional Continuous Normalizing Flows (CNFs). Our evaluations confirmed NodeFlow’s exceptional capability in managing high-dimensional multivariate probabilistic regression tasks, effectively aligning with benchmarks for tasks with one-dimensional targets. Ablation studies elucidated the critical roles of the NODE and CNF components in NodeFlow’s architecture, enhancing feature processing and complex distribution modeling, respectively. Moreover, NodeFlow emerges as a robust solution for advanced modeling and uncertainty quantification in regression tasks, adeptly balancing performance with computational efficiency. It not only establishes a significant presence in the domain of probabilistic regression but also lays a foundation for future advancements in machine learning interpretability and robustness. The differentiability of NodeFlow’s architecture is particularly conducive to further research in interpretability techniques, including counterfactual explanations, feature attribution, and adversarial example generation, promising substantial contributions to the field’s evolution.

## Figures and Tables

**Table 1:** Benchmark for *univariate probabilistic* regression problem with tabular data using negative log-likelihood (NLL) as the metric. The best results are marked by **bold text**, and the second best results are underlined.

Dataset	Deep. Ens.	CatBoost	NGBoost	RoNGBa	PGBM	TreeFlow	NodeFlow
Concrete	3.06 ± 0.18	3.06 ± 0.13	3.04 ± 0.17	2.94 ± 0.18	**2.75 ± 0.21**	3.02 ± 0.15	3.15 ± 0.21
Energy	1.38 ± 0.22	1.24 ± 1.28	0.60 ± 0.45	**0.37 ± 0.28**	1.74 ± 0.04	0.85 ± 0.35	0.90 ± 0.25
Kin8nm	**−1.20 ± 0.02**	−0.63 ± 0.02	−0.49 ± 0.02	−0.60 ± 0.03	−0.54 ± 0.04	−1.03 ± 0.06	−1.10 ± 0.05
Naval	**−5.63 ± 0.05**	−5.39 ± 0.04	−5.34 ± 0.04	−5.49 ± 0.04	−3.44 ± 0.04	−5.54 ± 0.16	−5.45 ± 0.08
Power	2.79 ± 0.04	2.72 ± 0.12	2.79 ± 0.11	2.65 ± 0.08	**2.60 ± 0.02**	2.65 ± 0.06	2.62 ± 0.05
Protein	2.83 ± 0.02	2.73 ± 0.07	2.81 ± 0.03	2.76 ± 0.03	2.79 ± 0.01	**2.02 ± 0.02**	2.04 ± 0.04
Wine	0.94 ± 0.12	0.93 ± 0.08	0.91 ± 0.06	0.91 ± 0.08	0.97 ± 0.20	**−0.56 ± 0.62**	−0.21 ± 0.28
Yacht	1.18 ± 0.21	0.41 ± 0.39	0.20 ± 0.26	1.03 ± 0.44	**0.05 ± 0.28**	0.72 ± 0.40	0.79 ± 0.55
Year MSD	3.35 ± NA	3.43 ± NA	3.43 ± NA	3.46 ± NA	3.61 ± NA	3.27 ± NA	**3.09 ± NA**

**Table 2:** Benchmark for *multivariate probabilistic* regression problem with tabular data using negative log-likelihood (NLL) as the metric. The best results are marked by **bold text**, and the second best results are underlined.

Dataset	Ind. NGBoost	NGBoost	TreeFlow	NodeFlow
Parkinsons	6.86	5.85	5.26	**5.06**
Scm20d	94.40	94.81	93.41	**91.98**
Wind	−0.65	−0.67	−2.57	**−3.20**
Energy	166.90	175.80	180.00	**163.86**
USflight	9.56	8.57	7.49	**7.38**
Ocean.	7.74	**7.73**	7.84	7.81

**Table 3:** Benchmark for *univariate point prediction* regression problem with tabular data using Root-Mean-Square Error (RMSE). Note that for TreeFlow and NodeFlow, we used the RMSE@2 metric, which is more relevant. The best results are marked by **bold text**, and the second best results are underlined.

Dataset	Deep. Ens.	CatBoost	NGBoost	RoNGBa	PGBM	TreeFlow (@2)	NodeFlow(@2)
Concrete	6.03 ± 0.58	5.21 ± 0.53	5.06 ± 0.61	4.71 ± 0.61	**3.97 ± 0.76**	5.41 ± 0.71	5.51 ± 0.66
Energy	2.09 ± 0.29	0.57 ± 0.06	0.46 ± 0.06	**0.35 ±0.07**	**0.35 ± 0.06**	0.65 ± 0.12	0.70 ± 0.40
Kin8nm	0.09 ± 0.00	0.14 ± 0.00	0.16 ± 0.00	0.14 ± 0.00	0.13 ± 0.01	0.10 ± 0.01	**0.08 ± 0.00**
Naval	**0.00 ± 0.00**	**0.00 ± 0.00**	**0.00 ± 0.00**	**0.00 ± 0.00**	**0.00 ± 0.00**	**0.00 ± 0.00**	**0.00 ± 0.00**
Power	4.11 ± 0.17	3.55 ± 0.27	3.70 ± 0.22	3.47 ± 0.19	**3.35 ± 0.15**	3.79 ± 0.25	3.94 ± 0.16
Protein	4.71 ± 0.06	3.92 ± 0.08	4.33 ± 0.03	4.21 ± 0.06	3.98 ± 0.06	**3.01 ± 0.06**	4.32 ± 0.03
Wine	0.64 ± 0.04	0.63 ± 0.04	0.62 ± 0.04	0.62 ± 0.05	0.60 ± 0.05	**0.41 ± 0.09**	0.44 ± 0.03
Yacht	1.58 ± 0.48	0.82 ± 0.40	**0.50 ± 0.20**	0.90 ± 0.35	0.63 ± 0.21	0.75 ± 0.26	1.18 ± 0.47
Year MSD	8.89 ± NA	8.99 ± NA	8.94 ± NA	9.14 ± NA	9.09 ± NA	**8.64 ± NA**	8.84 ± NA

**Table 4:** Ablation study of the *feature representation* component in terms of negative log-likelihood (NLL), Continuous Ranked Probability Score (CRPS), and Root-Mean-Square Error at 2 (RMSE@2) metrics.

Dataset	NLL			CRPS			RMSE		
	CNF	CNF + MLP	NodeFlow	CNF	CNF + MLP	NodeFlow	CNF	CNF + MLP	NodeFlow
Concrete	3.24 ± 0.28	**3.15 ± 0.13**	**3.15 ± 0.21**	3.80 ± 1.33	3.39 ± 0.34	**2.80 ± 0.34**	7.16 ± 2.22	6.43 ± 0.54	**5.51 ± 0.66**
Energy	2.90 ± 0.45	2.43 ± 0.31	**0.90 ± 0.25**	2.73 ± 1.45	1.73 ± 0.77	**0.35 ± 0.14**	4.90 ± 2.41	3.26 ± 1.26	**0.70 ± 0.40**
Kin8nm	−0.66 ± 0.12	−0.86 ± 0.07	**−1.10 ± 0.05**	0.07 ± 0.01	0.06 ± 0.00	**0.04 ± 0.00**	0.14 ± 0.02	0.11 ± 0.01	**0.08 ± 0.00**
Naval	−3.42 ± 0.34	−3.55 ± 0.21	**−5.45 ± 0.08**	0.01 ± 0.00	**0.00 ± 0.00**	**0.00 ± 0.00**	0.01 ± 0.00	0.01 ± 0.00	**0.00 ± 0.00**
Power	2.92 ± 0.24	2.90 ± 0.26	**2.62 ± 0.05**	2.59 ± 1.00	2.61 ± 1.15	**1.95 ± 0.06**	4.69 ± 1.71	4.77 ± 1.94	**3.94 ± 0.16**
Protein	2.57 ± 0.03	2.56 ± 0.02	**2.04 ± 0.04**	2.69 ± 0.04	2.67 ± 0.03	**1.75 ± 0.03**	5.88 ± 0.11	5.81 ± 0.10	**4.32 ± 0.03**
Wine	0.07 ± 0.62	0.34 ± 0.63	**−0.21 ± 0.28**	0.36 ± 0.04	0.37 ± 0.04	**0.34 ± 0.02**	0.54 ± 0.14	0.61 ± 0.14	**0.44 ± 0.09**
Yacht	1.92 ± 1.67	1.35 ± 1.82	**0.79 ± 0.55**	2.45 ± 3.06	1.26 ± 2.35	**0.50 ± 0.19**	5.06 ± 5.42	2.71 ± 4.33	**1.18 ± 0.47**

**Table 5:** Ablation study of the *probabilistic modeling* component in terms of negative log-likelihood (NLL), Continuous Ranked Probability Score (CRPS), and Root-Mean-Square Error at 2 (RMSE@2) metrics.

Dataset	NLL			CRPS			RMSE		
	NodeGauss	NodeGMM	NodeFlow	NodeGauss	NodeGMM	NodeFlow	NodeGauss	NodeGMM	NodeFlow
Concrete	3.13 ± 0.39	**3.03 ± 0.18**	3.15 ± 0.21	8.54 ± 0.49	9.04 ± 0.49	**2.80 ± 0.34**	15.52 ± 0.86	16.08 ± 0.86	**5.51 ± 0.66**
Energy	1.84 ± 0.23	1.70 ± 0.21	**0.90 ± 0.25**	5.16 ± 0.27	5.59 ± 0.27	**0.35 ± 0.14**	9.53 ± 0.41	9.94 ± 0.41	**0.70 ± 0.40**
Kin8nm	−0.90 ± 0.07	−0.97 ± 0.06	**−1.10 ± 0.05**	0.14 ± 0.00	0.15 ± 0.00	**0.04 ± 0.00**	0.18 ± 0.01	0.22 ± 0.01	**0.08 ± 0.00**
Naval	−4.91 ± 0.29	−4.95 ± 0.15	**−5.45 ± 0.08**	0.01 ± 0.00	0.01 ± 0.00	**0.00 ± 0.00**	0.01 ± 0.00	0.01 ± 0.00	**0.00 ± 0.00**
Power	2.84 ± 0.05	2.76 ± 0.04	**2.62 ± 0.05**	8.88 ± 0.12	9.59 ± 0.12	**1.95 ± 0.06**	16.10 ± 0.22	16.88 ± 0.23	**3.94 ± 0.16**
Protein	2.84 ± 0.07	2.36 ± 0.12	**2.04 ± 0.04**	3.39 ± 0.02	3.39 ± 0.03	**1.75 ± 0.03**	6.03 ± 0.06	7.40 ± 0.36	**4.32 ± 0.03**
Wine	0.97 ± 0.08	0.51 ± 0.37	**−0.21 ± 0.28**	0.45 ± 0.03	0.45 ± 0.03	**0.34 ± 0.02**	0.82 ± 0.05	0.59 ± 0.16	**0.44 ± 0.09**
Yacht	2.26 ± 0.72	1.84 ± 0.63	**0.79 ± 0.55**	6.67 ± 1.52	6.62 ± 1.58	**0.50 ± 0.19**	14.19 ± 3.02	14.26 ± 2.95	**1.18 ± 0.47**

**Table 6:** Comparative analysis of training duration for NodeFlow and ablation study approaches.

Dataset	CNF	CNF + MLP	NodeGauss	NodeGMM	NodeFlow
Concrete	335.23 ± 64.91 s	431.65 ± 232.73 s	43.82 ± 15.28 s	25.20 ± 9.74 s	482.69 ± 127.31 s
Energy	70.63 ± 6.34 s	80.83 ± 7.33 s	23.25 ± 7.35 s	15.48 ± 6.36 s	687.24 ± 99.62 s
Kin8nm	137.19 ± 9.76 s	169.22 ± 40.49 s	45.72 ± 13.31 s	55.14 ± 16.32 s	308.89 ± 61.57 s
Naval	213.13 ± 61.62 s	228.93 ± 20.99 s	56.22 ± 20.75 s	47.74 ± 27.42 s	2413.23 ± 649.67 s
Power	141.333 ± 12.30 s	180.81 ± 17.90 s	40.19 ± 15.56 s	43.93 ± 15.51 s	1360.29 ± 192.94 s
Protein	373.255 ± 40.39 s	417.45 ± 52.54 s	217.13 ± 22.18 s	224.45 ± 63.75 s	3018.98 ± 616.95 s
Wine	352.964 ± 69.65 s	353.93 ± 67.75 s	26.82 ± 10.80 s	11.92 ± 6.41 s	614.85 ± 136.68 s
Yacht	203.561 ± 117.80 s	259.64 ± 135.60 s	19.50 ± 10.33 s	13.31 ± 4.60 s	567.44 ± 216.81 s

**Figure A1:**
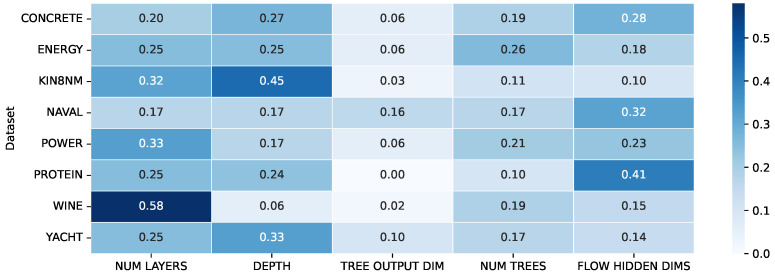
Hyperparameter importance analysis in the NodeFlow tuning process. Importance scores for each dataset and searched hyperparameter were calculated using the fANOVA Hyperparameter Importance Evaluation algorithm, with the highest scores underlining their pivotal role in the optimization process.

## Data Availability

The data presented in this study are openly available in https://github.com/pfilo8/NodeFlow (accessed date: 28 May 2024).

## Appendix A. Datasets

In this section, we delve into the details of the datasets used in our study to validate the capabilities of NodeFlow empirically. These datasets are the standard in assessing method effectiveness and were chosen to evaluate NodeFlow’s performance across various domains and to demonstrate its versatility in addressing complex probabilistic regression tasks. Table A1 furnishes comprehensive details on the datasets employed, encompassing the number of data points (N), the quantity of cross-validation (CV) splits or test dataset observations, along with the feature dimensionality (D) and target dimensionality (P).

What is important and different from the reference methods is that the datasets utilized in our study were scaled to the range (−1,1), encompassing both the features and target variables. This crucial preprocessing step was undertaken with a specific purpose in mind—to enhance the stability of the learning process within the neural network framework. By scaling both the features and targets to this common range, we aimed to mitigate potential issues related to the magnitude of data values, which can impact the convergence and performance of neural networks during training.

It is worth noting that the tree-based methods with which we compared NodeFlow did not require the extensive scaling of both features and targets as they inherently possess a scale-invariance property. This characteristic stems from the way decision trees partition the feature space, making them less sensitive to variations in feature and target scales and thereby obviating the need for such preprocessing.

**Table A1:** An overview of the datasets employed in our study to assess the performance of NodeFlow. The table includes information on the number of data points (N), the number of cross-validation (CV) splits or observations in the test dataset, feature dimensionality (D), and target dimensionality (P).

Dataset	N	CV Splits/Ntest	D	P
Concrete	1030	20 CV	8	1
Energy	768	20 CV	8	1
Kin8nm	8192	20 CV	8	1
Naval	11,934	20 CV	16	1
Power	9568	20 CV	4	1
Protein	45,730	5 CV	9	1
Wine	1588	20 CV	11	1
Yacht	308	20 CV	6	1
Year MSD	515,345	1 CV	90	1
Parkinsons	4112	1763	16	2
scm20d	7173	1793	61	16
WindTurbine	4000	1000	8	6
Energy	57,598	14,400	32	17
usFlight	500,000	200,000	8	2
Oceanographic	373,227	41,470	9	2

## Appendix B. Implementation Details

The research methodology adhered to the standard practices characteristic of machine learning projects. All models under consideration were implemented using Python 3.8, leveraging the deep learning library PyTorch. The training employed the usage of the PyTorch Lightning framework. We used the following infrastructure for the experiments: Intel(R) Xeon(R) Silver 4108 32-Core CPU, 4 NVIDIA GeForce GTX 1080 Ti GPUs, and 126 GB RAM.

In our research paper, we employed a Hyperband Pruner [25] as the hyperparameter search method to optimize our machine learning models. Hyperband Pruner is a highly efficient technique that focuses on identifying promising hyperparameter configurations while discarding less promising ones. To explore the hyperparameter space effectively, we uniformly sampled parameters within the specified ranges, as detailed in Table A2. Each dataset underwent a comprehensive search process, with each fold requiring a maximum duration of three hours. This approach allowed us to tune our models efficiently and select the best-performing hyperparameters, ultimately enhancing the predictive capabilities of our machine learning algorithms.

Based on the results of the hyperparameter search, we conducted a comprehensive analysis to evaluate the significance of hyperparameters in the tuning process. To assess this, we employed the fANOVA Hyperparameter Importance Evaluation algorithm [26], which involves fitting a random forest regression model to predict the objective values of successfully completed trials based on their parameter configurations. The outcomes of this analysis are illustrated in Figure A1.

As depicted in the figure, three particular hyperparameters were identified as crucial in our hyperparameter tuning process. These critical hyperparameters are the number of layers and the depth of the trees within the NODE (Neural Oblivious Decision Ensemble) component and the dimensionality of the hidden layers within the CNF (Conditional Continuous Normalizing Flow) component. These specific hyperparameters played a pivotal role in influencing the model’s performance and its ability to generalize effectively. Interestingly, the hyperparameter related to the output dimension of the NODE’s tree did not exhibit a significant impact on the results.

**Table A2:** Comprehensive overview of the hyperparameters employed in our research for optimizing the NodeFlow method. The hyperparameter ranges and settings for various datasets are detailed, allowing for a clear understanding of the tuning process.

Dataset	num layers	depth	tree output dim	num trees	flow hidden dims	n epochs	# of iterations
concrete	1–8	1–7	1–3	100–600	[4,4], [8,8], [16,16], [32,32]	400	400
energy	1–8	1–6	1–3	100–600	[4,4], [8,8], [16,16], [32,32]	400	300
kin8nm	1–8	1–6	1–3	100–600	[4,4], [8,8], [16,16], [32,32]	100	100
naval	1–8	1–6	1–3	100–600	[4,4], [8,8], [16,16], [32,32]	300	100
power	1–8	1–6	1–3	100–600	[4,4], [8,8], [16,16], [32,32]	200	100
protein	1–8	1–6	1–3	100–600	[4,4], [8,8], [16,16], [32,32]	100	100
wine	1–8	1–6	1–3	100–600	[4,4], [8,8], [16,16], [32,32]	400	500
yacht	1–8	1–6	1–3	100–500	[4,4], [8,8], [16,16], [32,32]	400	400
year msd	6	2, 4	1	100, 300	[4,4], [8,8], [16,16], [32,32]	10	16

## References

[1] Borisov V., Leemann T., Seßler K., Haug J., Pawelczyk M., Kasneci G. (2021). Deep Neural Networks and Tabular Data: A Survey. arXiv.

[2] Grinsztajn L., Oyallon E., Varoquaux G. Why do tree-based models still outperform deep learning on typical tabular data?. Proceedings of the Advances in Neural Information Processing Systems 35: Annual Conference on Neural Information Processing Systems 2022, NeurIPS 2022.

[3] Chen T., Guestrin C. XGBoost: A Scalable Tree Boosting System. Proceedings of the 22nd ACM SIGKDD International Conference on Knowledge Discovery and Data Mining.

[4] Prokhorenkova L.O., Gusev G., Vorobev A., Dorogush A.V., Gulin A. CatBoost: Unbiased boosting with categorical features. Proceedings of the Advances in Neural Information Processing Systems 31: Annual Conference on Neural Information Processing Systems 2018, NeurIPS 2018.

[5] Ke G., Meng Q., Finley T., Wang T., Chen W., Ma W., Ye Q., Liu T. LightGBM: A Highly Efficient Gradient Boosting Decision Tree. Proceedings of the Advances in Neural Information Processing Systems 30: Annual Conference on Neural Information Processing Systems 2017.

[6] Popov S., Morozov S., Babenko A. Neural Oblivious Decision Ensembles for Deep Learning on Tabular Data. Proceedings of the 8th International Conference on Learning Representations, ICLR 2020.

[7] Abutbul A., Elidan G., Katzir L., El-Yaniv R. (2020). DNF-Net: A Neural Architecture for Tabular Data. arXiv.

[8] Gorishniy Y., Rubachev I., Khrulkov V., Babenko A., Ranzato M., Beygelzimer A., Dauphin Y.N., Liang P., Vaughan J.W. Revisiting Deep Learning Models for Tabular Data. Proceedings of the Advances in Neural Information Processing Systems 34: Annual Conference on Neural Information Processing Systems 2021, NeurIPS 2021.

[9] Duan T., Anand A., Ding D.Y., Thai K.K., Basu S., Ng A.Y., Schuler A. NGBoost: Natural Gradient Boosting for Probabilistic Prediction. Proceedings of the 37th International Conference on Machine Learning, ICML 2020, PMLR.

[10] Sprangers O., Schelter S., de Rijke M. Probabilistic Gradient Boosting Machines for Large-Scale Probabilistic Regression. Proceedings of the KDD ’21: The 27th ACM SIGKDD Conference on Knowledge Discovery and Data Mining.

[11] Malinin A., Prokhorenkova L., Ustimenko A. Uncertainty in Gradient Boosting via Ensembles. Proceedings of the 9th International Conference on Learning Representations, ICLR 2021.

[12] Wielopolski P., Zięba M. (2023). TreeFlow: Going Beyond Tree-Based Parametric Probabilistic Regression. ECAI 2023.

[13] Ren L., Sun G., Wu J. (2019). RoNGBa: A Robustly Optimized Natural Gradient Boosting Training Approach with Leaf Number Clipping. arXiv.

[14] Arik S.Ö., Pfister T. (2021). TabNet: Attentive Interpretable Tabular Learning. Proceedings of the Thirty-Fifth AAAI Conference on Artificial Intelligence, AAAI 2021, Thirty-Third Conference on Innovative Applications of Artificial Intelligence, IAAI 2021, The Eleventh Symposium on Educational Advances in Artificial Intelligence, EAAI 2021.

[15] Somepalli G., Goldblum M., Schwarzschild A., Bruss C.B., Goldstein T. (2021). SAINT: Improved Neural Networks for Tabular Data via Row Attention and Contrastive Pre-Training. arXiv.

[16] Lakshminarayanan B., Pritzel A., Blundell C. Simple and Scalable Predictive Uncertainty Estimation using Deep Ensembles. Proceedings of the Advances in Neural Information Processing Systems 30: Annual Conference on Neural Information Processing Systems 2017.

[17] Gal Y., Ghahramani Z., Balcan M., Weinberger K.Q. (2016). Dropout as a Bayesian Approximation: Representing Model Uncertainty in Deep Learning. JMLR Workshop and Conference Proceedings, Proceedings of the 33nd International Conference on Machine Learning, ICML 2016, New York, NY, USA, 19–24 June 2016.

[18] Hernández-Lobato J.M., Adams R.P., Bach F.R., Blei D.M. (2016). Probabilistic Backpropagation for Scalable Learning of Bayesian Neural Networks. JMLR Workshop and Conference Proceedings, Proceedings of the 32nd International Conference on Machine Learning, ICML 2015, Lille, France, 6–11 July 2015.

[19] Peters B., Niculae V., Martins A.F.T., Korhonen A., Traum D.R., Màrquez L. (2019). Sparse Sequence-to-Sequence Models. Proceedings of the 57th Conference of the Association for Computational Linguistics, ACL 2019.

[20] Yang G., Huang X., Hao Z., Liu M., Belongie S.J., Hariharan B. PointFlow: 3D Point Cloud Generation With Continuous Normalizing Flows. Proceedings of the 2019 IEEE/CVF International Conference on Computer Vision, ICCV 2019.

[21] Sendera M., Tabor J., Nowak A., Bedychaj A., Patacchiola M., Trzcinski T., Spurek P., Zieba M. Non-Gaussian Gaussian Processes for Few-Shot Regression. Proceedings of the Advances in Neural Information Processing Systems 34: Annual Conference on Neural Information Processing Systems 2021, NeurIPS 2021.

[22] Grathwohl W., Chen R.T.Q., Bettencourt J., Sutskever I., Duvenaud D. FFJORD: Free-Form Continuous Dynamics for Scalable Reversible Generative Models. Proceedings of the 7th International Conference on Learning Representations, ICLR 2019.

[23] Carlisle M. (2019). Racist Data Destruction?. https://medium.com/@docintangible/racist-data-destruction-113e3eff54a8.

[24] McInnes L., Healy J. (2018). UMAP: Uniform Manifold Approximation and Projection for Dimension Reduction. arXiv.

[25] Li L., Jamieson K.G., DeSalvo G., Rostamizadeh A., Talwalkar A. (2017). Hyperband: A Novel Bandit-Based Approach to Hyperparameter Optimization. J. Mach. Learn. Res..

[26] Hutter F., Hoos H.H., Leyton-Brown K. An Efficient Approach for Assessing Hyperparameter Importance. Proceedings of the 31th International Conference on Machine Learning, ICML 2014.

